# A New Species of *Boulenophrys* (Anura, Megophryidae) from Northern Jiangxi, China †

**DOI:** 10.3390/ani15213197

**Published:** 2025-11-03

**Authors:** Deming Shen, Haiying Zhou, Kevin R. Messenger, Hina Amin, Zhenyu Wang, Jun Xu, Shi Xu, Yankuo Li

**Affiliations:** 1 College of Life Sciences, Jiangxi Normal University, Nanchang 330022, China; 2 Laboratory of Herpetology and Applied Conservation, Nanjing Forestry University, Nanjing 210037, China; kevinrmessenger@gmail.com; 3 School of Marine Biology and Fisheries, Hainan University, Haikou 570228, China; 4 Administration of Lushan National Nature Reserve, Jiujiang 332900, China

**Keywords:** mount lushan, Asian horned toads, integrative taxonomy, biodiversity, *Boulenophrys lushanensis* sp. nov

## Abstract

**Simple Summary:**

The genus *Boulenophrys* Fei, Ye & Jiang, 2016 (Megophryinae) currently comprises 77 recognized species and represents one of the most species-rich anuran genera in Asia. In this study, we described a population of Asian horned toads on Mount Lushan that had long been misidentified as other species. We integrated data on mitochondrial genes, morphology and advertisement calls. The combined evidence firmly supports the Lushan population as a distinct species. Recognizing this hidden diversity not only enriches our understanding of biodiversity in Jiangxi Province but also helps guide efforts to protect these unique amphibians and the special mountain streams they call home.

**Abstract:**

A new species of the genus *Boulenophrys* (Anura, Megophryidae) is described from Mount Lushan, Jiangxi Province, China, long considered to be *Boulenophrys boettgeri*. Phylogenetic analyses based on mitochondrial COI and 16S rRNA genes show that the new species forms a sister clade to *B. jinggangensis*, with an uncorrected COI p-distance of 3.7%, confirming its status as a distinct species. Morphologically, it differs from all known congeners by a unique combination of characters, and from its closest relative *B. jinggangensis* by having the following traits: (1) larger adult body size (adult males SVL 42.7–44.7 mm; adult females 46.0–50.3 mm); (2) heels not meeting when hindlimbs are flexed at right angles to the body axis; (3) smaller horn-like tubercle on the upper eyelid; and (4) relative finger lengths (I < II < IV < III). Acoustically, the new species differs markedly from *B. jinggangensis* in nearly all parameters of its advertisement calls. This discovery increases the known species diversity of *Boulenophrys* in Jiangxi Province and provides baseline data for further biodiversity conservation efforts in the Mount Lushan region.

## 1. Introduction

The genus *Boulenophrys* Fei, Ye & Jiang, 2016 (Megophryinae) currently comprises 77 recognized species and represents one of the most species-rich anuran genera in Asia (Table S1). Its members are widely distributed across the Himalayas, northeastern India, southern China, and Southeast Asia [1,2,3]. Despite its broad range, recent integrative studies have revealed extensive cryptic diversity within *Boulenophrys*, much of which is not discernible through morphology alone [4,5,6]. These findings have spurred taxonomic revisions within the subfamily Megophryinae, with Lyu et al. [7] proposing a ten-genus system that affirms *Boulenophrys* as the most species-rich lineage. In Jiangxi Province, known *Boulenophrys* species are primarily described from the Luoxiao Mountains along the Hunan border and the Wuyi Mountains bordering Fujian [8]. However, the northern part of Jiangxi, including the topographically isolated granitic massif of Mount Lushan in the Yangtze River Basin, has received limited herpetological attention. This suggests that the species diversity of *Boulenophrys* in this region may still be underestimated.

The *Boulenophrys* population from Mount Lushan was first recorded by Boettger in 1894. The specimen, collected near Jiujiang City, was classified as *Leptobrachium monticola* [9]. Later, Boulenger described the species based on material from Guadun (Fujian) and named it *Leptobrachium boettgeri* in 1899 [10]. Consequently, the Lushan population was placed under *Boulenophrys boettgeri*, a name that was also subsequently applied to populations across various regions for an extended period [11,12,13]. However, recent large-scale sampling and taxonomic revisions across localities have indicated that records of *Boulenophrys boettgeri* from various regions may involve misidentifications. Specifically, specimens from at least Guangdong and Guangxi Provinces likely represent distinct species as noted by Lyu et al. [7]. The Lushan population was then considered part of the distribution of *Boulenophrys jinggangensis*, though no further detailed genetic or morphological analyses were provided, leaving its taxonomic identity unresolved.

Herpetological surveys on Mount Lushan since 2021 have led to the collection of several horned toad specimens. Morphological examinations revealed that these individuals could not be classified under any known species from the region. Although they share a superficial resemblance to *Boulenophrys jinggangensis*, the Lushan specimens exhibit distinct differences in a combination of morphological characters and strikingly differ in call rate from *B. jinggangensis*. To further clarify their taxonomic status, we sequenced mitochondrial gene fragments (16S and COI), conducted phylogenetic analyses, and performed detailed morphological comparisons and call analyses. Integrative evidence supports the recognition of the Lushan population as a distinct evolutionary lineage. Based on this evidence, we formally describe it herein as a new species.

## 2. Materials and Methods

### 2.1. Sampling

**Figure 1:**
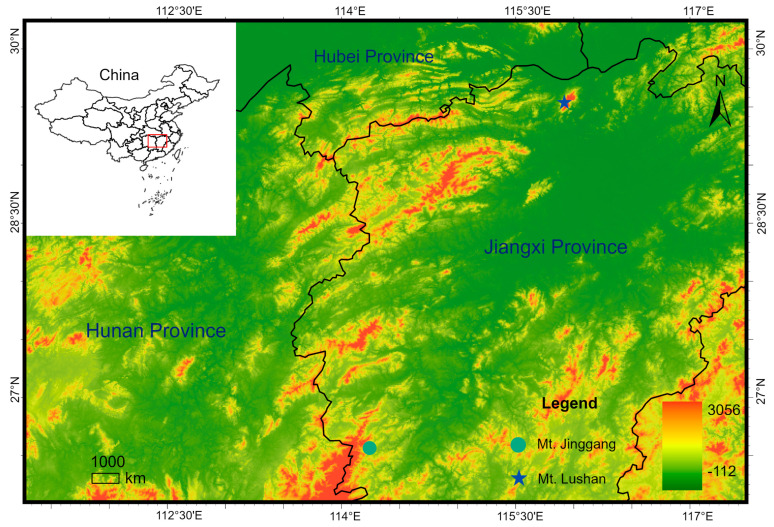
Sampling and recording localities of *Boulenophrys lushanensis* sp. nov. from Mount Lushan, Lushan City, Jiangxi Province, China, and *B. jinggangensis* from Mount Jinggang, Jinggangshan City, Jiangxi Province, China.

Fieldwork was conducted from 15 to 30 April and 1 to 15 June 2021, at Mount Lushan (Figure 1), China. Nine adult specimens were collected. After capture, the specimens were euthanized with tricaine methanesulfonate (MS-222; Sigma-Aldrich, St. Louis, MO, USA). Liver or thigh muscle tissues were taken for molecular experiments, preserved in 95% ethanol, and stored at −70 °C. Whole specimens were fixed in 75% ethanol. All specimens and tissue samples were deposited in the Animal Museum of Life Sciences College of Jiangxi Normal University (JXNU), Nanchang, Jiangxi Province, China.

### 2.2. Morphological Analyses

Morphological terminology and character definitions followed Lyu et al. [7] and subsequent taxonomic revisions of *Boulenophrys*. External measurements were taken using a digital caliper (Neiko 01407A; Neiko, West Chester, OH, USA) to the nearest 0.1 mm. The following measurements were recorded: snout–vent length (SVL), head length (HL), head width (HW), snout length (SL), internasal distance (IND), interorbital distance (IOD), eye diameter (ED), tympanum diameter (TD), tympanum–eye distance (TED), hand length (HNL), forearm length (FAL), tibial length (TL), and foot length (FTL). Measurement definitions followed standard practice, such as HL from the snout tip to the jaw articulation, and TL from the outer surface of the flexed knee to the heel. Sex was determined by the presence of secondary sexual characteristics, including internal vocal sac openings or nuptial pads in males, and by direct observation of advertisement calls where possible Fei et al. [12]. The presence or absence of nuptial pads was verified under a dissecting microscope. Comparative morphological data for congeners were compiled from original species descriptions and taxonomic revisions available in the literature, ensuring consistency in character definitions and measurement standards.

### 2.3. DNA Sequencing and Molecular Analyses

Genomic DNA was extracted from approximately 0.1 g of liver or muscle tissue using the Universal Genomic DNA Kit (CWBIO, Beijing, China), following the manufacturer’s protocol for animal tissue. Two mitochondrial gene fragments, the partial 16S ribosomal RNA coding sequence (16S rRNA, 536 bp) and the partial cytochrome c oxidase subunit I gene (COI, 632 bp), were amplified. For 16S, primers L3975 (5′-CGCCTGTTTACCAAAAACAT-3′) and H4551 (5′-CCGGTCTGAACTCAGATCACGT-3′) were used; for COI, primers DGLCO (5′-GGTCAACAAATCATAAAGAYATYGG-3′) and dgHCO (5′-AAACTTCAGGGTGACCAAARAAYCA-3′) were used, following Liu et al. [5]. PCRs were performed in a 50 μL reaction volume containing 25 μL of 2× Taq PCR MasterMix (Tiangen, Beijing, China), 2 μL of each primer (10 μM), approximately 100 ng of genomic DNA, and nuclease-free water. The cycling profile consisted of an initial denaturing step at 95 °C for 4 min; 36 cycles of 95 °C for 30 s, annealing at 52 °C for 16S or 47 °C for COI for 40 s, and extension at 72 °C for 70 s; followed by a final extension at 72 °C for 10 min. Negative controls were included to check for contamination. PCR products were purified and sequenced bidirectionally using an ABI 3730 automated DNA sequencer (Shanghai DNA BioTechnologies Co., Ltd., Shanghai, China). All sequences were verified by comparing the forward and reverse reads to ensure no double peaks were present. Sequences were assembled and edited in Geneious Prime 2025. New sequences were deposited in GenBank (Table S2).

For the phylogenetic analyses, sequences of 77 *Boulenophrys* species were obtained from GenBank, except for *B. changyangensis*, *B. dalaolingensis* and *B. gutu* due to unavailable data. Two outgroup species, *Xenophrys glandulosa* and *X. mangshanensis*, were also included (Table S2). All sequences were aligned using the MUSCLE v3.8.31 algorithm with default parameters [14]. Phylogenetic analyses were conducted based on a concatenated mitochondrial dataset comprising COI and 16S rRNA genes. The final alignment was 1333 bp in length, including 482 parsimony-informative sites, with 8.6% missing data.

Both Maximum Likelihood (ML) and Bayesian Inference (BI) methods were employed to construct phylogenetic trees [15,16]. The ML analysis was performed in IQ-TREE v3.0.1, with the best-fit substitution model (TVM + F + R5) selected using ModelFinder. Branch support was assessed using 1000 ultrafast bootstrap replicates. BI analysis was conducted in MrBayes v3.2, using gene partitions defined by PartitionFinder 2 (16S: 1–626; COI: 627–1333) [17]. Markov Chain Monte Carlo (MCMC) sampling was run for 2,000,000 generations, with trees sampled every 1000 generations and the first 25% discarded as burn-in. Convergence was considered achieved when the average standard deviation of split frequencies dropped below 0.01.

### 2.4. Acoustic Analyses

Advertisement calls of the undescribed species were recorded from the holotype (JXNU 21042803) on 28 April 2021, at a distance of 0.6 m, and from another specimen (JXNU 21042802) and one unvouchered individual at a distance of 0.2 m, on Mount Lushan, Jiujiang City, Jiangxi Province, China (ambient temperature: 24.5 °C, relative humidity: 91%). For comparisons, advertisement calls of *Boulenophrys jinggangensis* were recorded from an unvouched specimen at a distance of 0.2 m on 9 July 2025 at its type locality, Mount Jinggang (26°33.11′ N, 114°9.29′ E; 845 m a.s.l.), Jinggangshan City, Jiangxi Province, China (ambient temperature: 22.4 °C, relative humidity: 88%). Digital recordings were made using a TASCAM DR05X recorder (TASCAM, Tokyo, Japan) (96 kHz sampling rate, 24-bit depth) with a built-in microphone. The acoustic analysis implemented a Python 3.9 pipeline using librosa [18] for audio processing, noisereduce for spectral denoising, and scipy.signal.find_peaks for pulse detection. Core procedures included amplitude-threshold segmentation to isolate call groups, spectral peak identification to count pulses (distinct sound units within a call group), and extraction of dominant frequency (the frequency with the highest energy) via 512-point Hann-windowed Fast Fourier Transform (FFT). All parameters were measured following Tapley et al. [19] standards.

## 3. Results

### 3.1. Phylogenetic Analyses and Genetic Divergence

**Figure 2:**
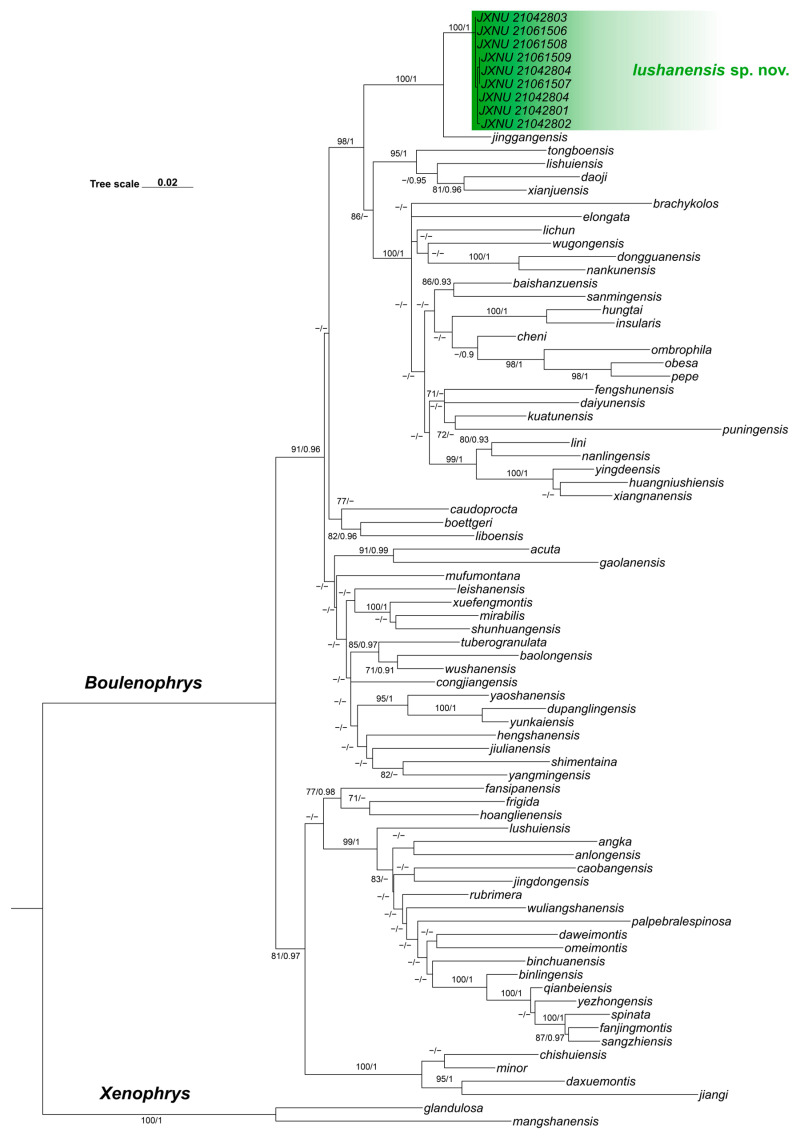
The Maximum Likelihood topology, inferred from partial DNA sequences of the mitochondrial 16S rRNA and COI genes. Bootstrap support values and Bayesian posterior probabilities are shown at the tree nodes. A dash (‘−’) denotes bootstrap support below 70 or Bayesian posterior probability below 0.9.

Phylogenetic trees reconstructed using Bayesian inference (BI) and Maximum Likelihood (ML) methods showed highly consistent topologies. In both analyses, the major nodes were well supported, corresponding to several principal species-groups within *Boulenophrys*, including *B. minor*, *B. omeimontis*, and *B. boettgeri* groups. Within *B. boettgeri* group, the clade containing *B. yunkaiensis*, *B. yaoshanensis*, and *B. gaolanensis* forms a sister taxon to the clade containing *B. boettgeri* (BS = 91; BPP = 0.96). Inside the *B. boettgeri* clade, *B. jinggangensis* and all samples collected from Mount Lushan each form a well-supported monophyletic clade (BS = 100; BPP = 1). These two clades form a well-supported sister clade (BS = 100; BPP = 1), representing a relatively early-diverging lineage. The ML tree is shown (Figure 2), with node support considered strong when bootstrap values (BS) ≥ 70 or Bayesian posterior probabilities (BPP) ≥ 0.90. Uncorrected COI p-distances within *Boulenophrys* range from 2.8–22.6% (Table S3), with genetic divergence between samples from Mount Lushan and its closest congener, *B. jinggangensis*, ranging from 3.4–3.7%, consistent with interspecific divergence.

In addition to these molecular findings, the Jiangxi Lushan lineage can be distinguished from all recognized congeners by a unique combination of morphological traits. Its advertisement calls are also distinct from those of its closest congener, *B. jinggangensis*. Based on phylogenetic, morphological, and acoustic evidence, we formally describe it below as a new species.

### 3.2. Taxonomic Account

*Boulenophrys lushanensis* Shen, Zhou & Li sp. nov.

Figure 3, Table 1

https://zoobank.org/D23C9445-19FE-47FA-9BC1-D6C3D08F5CB7 (accessed on 6 August 2025).

**Table 1:** Measurements (mm) of adult specimens in the type series of *B. lushanensis* sp. nov.

	JXNU 21042801	JXNU 21042802	JXNU 21042803	JXNU 21042804	JXNU 21061505	JXNU 21061506	JXNU 21061507	JXNU 21061508	JXNU 21061509
Sex	Female	Male	Male	Female	Female	Female	Male	Male	Male
SVL	50.2	44.7	43	50.3	46	46.3	41.1	40.5	42.7
HL	16.8	14.7	13.7	16.5	15.3	15.2	14	13.7	14.3
HW	16.7	14.4	14.2	16.9	15.6	15.8	14.4	14.7	14.6
SL	4.7	4.6	4.5	4.7	4.6	4.8	4.3	4.5	4.5
IND	4.2	3.6	3.6	3.9	3.8	3.9	3.7	3.8	3.6
IOD	4	3.5	3.8	4.1	3.5	3.6	3.7	3.5	3.4
ED	5.3	4.9	4.9	5.3	5	4.9	4.6	4.3	5.1
TD	3.4	3.3	2	2.9	2.6	2.6	1.7	2.2	2.6
TED	2.2	1.5	1.9	2.1	2	2.3	2	1.8	1.7
HNL	10.9	9.6	9.9	10.7	9.9	10.1	9.5	9.2	9.2
FAL	11.9	10.7	9.9	11.3	11.4	10.5	10.2	10.2	10.4
TL	21	18.7	18.2	20.2	20.3	19.5	18	18	17
FTL	19.8	17.8	17.7	18.8	18.4	17.6	16.8	16.9	16.6

**Figure 3:**
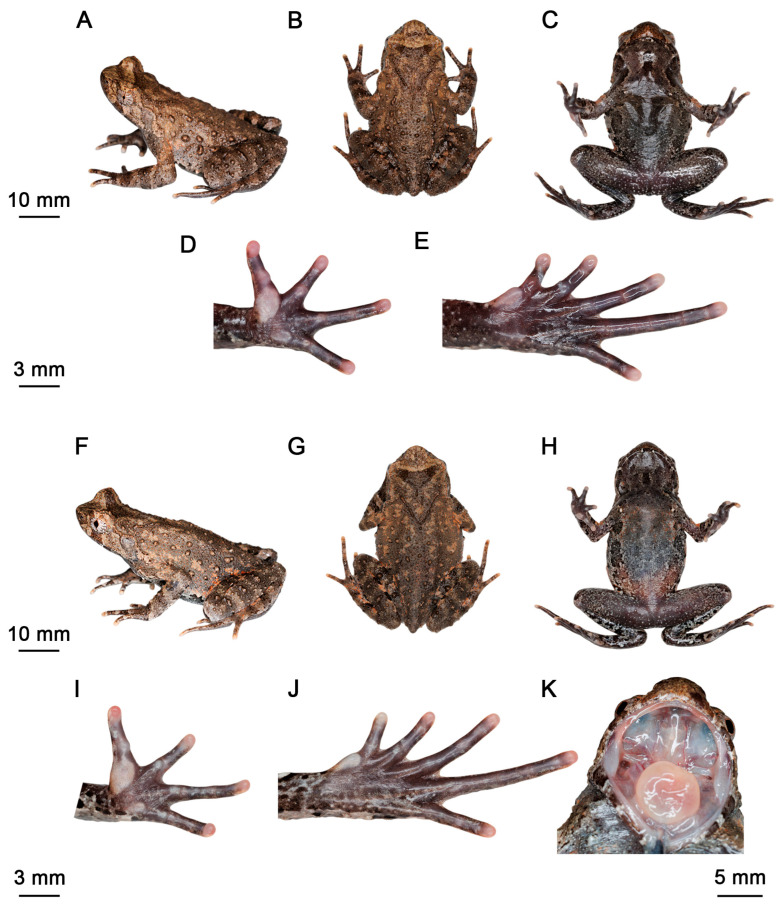
*Boulenophrys lushanensis* sp. nov. in life. Holotype (JXNU 21042803): (**A**) lateral view; (**B**) dorsal view; (**C**) ventral view; (**D**) volar view of left hand; (**E**) plantar view of left foot. Female paratype (JXNU 21061505): (**F**) lateral view; (**G**) dorsal view; (**H**) ventral view; (**I**) volar view of left hand; (**J**) plantar view of left foot; (**K**) edge of tongue.

Holotype. JXNU 21042803 (Figure 3A–E), adult male, collected by Deming Shen, Haiying Zhou, Yifeng Zhong, Jun Xu and Shi Xu on 28 April 2021 from Mount Lushan (29°31.87′ N, 115°55.87′ E; ca. 660 m a.s.l.), Lushan City, Jiujiang, Jiangxi, China.

Paratypes. (*n* = 8) JXNU 21042801, JXNU 21042804, adult females, and JXNU 21042802, adult male, collected by Deming Shen, Haiying Zhou, Yifeng Zhong, Jun Xu and Shi Xu on 28 April 2021 from Mount Lushan (29°32.56′ N, 115°57.58′ E; ca. 970 m a.s.l.); JXNU 21061505 (Figure 3F–K), JXNU 21061506, adult females, and JXNU 21061507, JXNU 21061508, JXNU21061509, adult males, collected by the same collectors on 15 June 2021 from the type locality (29°31.87′ N, 115°55.87′ E; ca. 660 m a.s.l.).

Etymology. The specific epithet *lushanensis* refers to Mount Lushan, the type locality of the new species in Jiangxi Province, China. We propose the English name “Lushan Horned Toad” and the Chinese name “庐山角蟾” (Lúshān Jiǎochán).

Diagnosis. This species can be distinguished from its congeners by the combination of following morphological characteristics: (1) adult males, SVL 40.5–44.7 mm (*n* = 5); adult females, SVL 46.0–50.2 mm (*n* = 4); (2) canthus rostralis well developed, tongue not notched posteriorly; (3) tympanum distinct; (4) vomerine ridge and vomerine teeth present; (5) hindlimbs slightly robust, heels not meeting when hindlimbs folded; when the hindlimbs is adpressed anteriorly along the body, tibiotarsal articulation reaches region between center of eye and anterior margin of tympanum; (6) outer margin of the upper eyelid bears a small, prominent, horn-like tubercle; (7) toes with narrow fringes and only rudimentary webbing; (8) relative finger lengths I < II < IV < III, a distinct subarticular tubercle is present at the base of each finger; (9) rough dorsal skin, a discontinuous V-shaped ridge is present on nape region; dorsal surface with short, irregular and weak ridges, with scattered tubercles and granules, dorsolateral ridges absent; several tubercles on flanks and dorsal hindlimbs; ventral skin finely granular; (10) dorsum light brown with irregular weak net-like brown markings; and dark brown triangular marking between eyes; dorsal limbs and digits with dark brown transverse bands; (11) dense nuptial spines on dorsal bases of fingers I and II in breeding adult males, subgular vocal sac present in males.

Description of holotype. Adult male, body size moderate, SVL 43.0 mm; head width slightly larger than head length, HW/HL 1.04; snout rounded in dorsal view, projecting, sloping backward to mouth in profile, protruding well beyond margin of lower jaw; top of head flat; eyes moderate in size, ED 0.36 of HL, pupil vertical, near diamond-shaped; nostril obliquely ovoid; canthus rostralis well developed; loreal region slightly oblique; internasal distance approximately equal to interorbital distance; tympanic region oblique, tympanum moderate in size, margin clear, upper margin in contact with supratympanic fold, TD/ED 0.41; large ovoid choanae at base of maxilla; vomerine ridge weak and vomerine teeth present, maxillary teeth present; margin of tongue rounded, not notched posteriorly; presence of subgular vocal sac.

Forearm length 0.23 of SVL; hand 0.23 of SVL; webbing absent between fingers; lateral fringes absent; relative finger length I < II < IV < III; tips of fingers slightly dilated, round; subarticular tubercles present and distinct, inner metacarpal tubercle observably enlarged; outer one slightly smaller; single nuptial pad bearing nuptial spines present on dorsal surface of first and second fingers, respectively.

Hindlimbs moderate in length; heels not meeting when flexed hindlimbs are held at right angles to the body axis; tibiotarsal articulation reaching the center of the eye when hindlimb are stretched forward along the body; tibiofibular length 0.42 of SVL, foot length 0.41 of SVL; relative toe length I < II < V < III < IV; tips of toes rounded and slightly dilated; toes with narrow lateral fringes and rudimentary webbing; subarticular tubercles distinct at the base of each toe; inner metatarsal tubercle long and ovoid; outer metatarsal tubercle absent; dorsal skin rough, with scattered tubercles, granules, and short, irregular, weak ridges; a discontinuous V-shaped ridge present on the nape region; dorsolateral ridges absent; several large rounded tubercles present on flanks and dorsal hindlimbs; a small, horn-like, prominent tubercle on the upper eyelid; supratympanic fold distinct, extending from the posterior corner of the eye to above the insertion of the forelimb; tympanum distinct; conical tubercles present on upper eyelid, loreal and temporal regions, upper lip, mandibular articulation, and area surrounding the cloaca; ventral skin finely granular, densely so on abdomen and ventral surfaces of limbs, and small, spinose tubercles surrounding the cloaca; pectoral gland small and distinct, located closer to axilla than to midline; a single femoral gland present, positioned approximately midway between knee and cloaca on posterior surface of thigh.

Coloration of holotype. In life, dorsum light yellowish-brown, with an incomplete dark brown triangular marking between the eyes, weak netlike brown markings scattered across the dorsum; dorsal surfaces of limbs brown, each bears several dark brown transverse bands; tubercles on the upper eyelids orange; a vertical dark brown stripe present below the eye, extending from the lower margin of the eye to the upper lip; surface of chest and throat bearing irregular black spots and white patches; ventral surface of limbs dark gray with irregular white and black patches; spines on tips of tubercles on ventral hindlimbs and area around cloaca blackish violet; ventral surfaces of hands and feet blackish violet; digits pale brown; tips of digits and inner metacarpal tubercles pinkish-white; subarticular tubercles and outer metatarsal tubercle gray-white; pectoral glands and femoral glands white; iris light yellowish-brown.

In preservative, dorsum faded from light yellowish-brown to greyish-brown; dark brown markings, specifically the triangular interorbital marking and transverse bands on the dorsal surfaces of the forelimbs and hindlimbs, have darkened, appearing black and becoming more conspicuous; ventral surfaces faded to greyish-white; white spots and blotches are now more prominent.

Variation. Morphometric data are given in Table 1. All paratypes conform to the holotype in key diagnostic characters but exhibit variation in coloration, with dorsum ranging from pale light yellowish-brown with yellowish-brown blotches (e.g., JXNU 21061506) to deeper brown with dark brown blotches (e.g., JXNU 21042802). Female paratypes also lack nuptial pads and keratinized spines on the forearms, features that are well developed in males.

**Figure 4:**
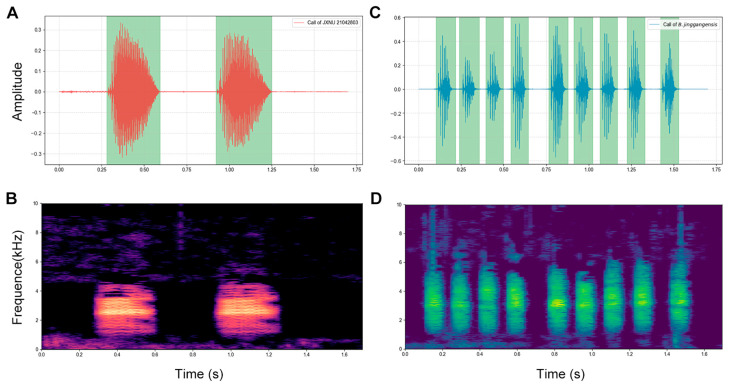
1.7 s advertisement call segments. *Boulenophrys lushanensis* sp. nov. (male holotype JXNU 21042803): (**A**) waveform; (**B**) spectrogram. *Boulenophrys jinggangensis* (unvouchered specimen from its type locality): (**C**) waveform; (**D**) spectrogram.

Advertisement call. Call descriptions are based on vocalizations of the male holotype JXNU 21042803. Spectrograms and waveforms are shown (Figure 4A,B). A total of seven call groups were analyzed. Each call had an average duration of 298.2 ± 18.1 ms (267.6–326.6 ms) and an average inter-call interval of 790.0 ± 124.3 ms (644.5–969.2 ms). Calls were repeated at a rate of 1.27 calls/s (constant repetition rate). Each call consisted of 47.8 ± 2.8 pulses (41–50 pulses). The dominant frequency averaged 2.56 ± 0.06 kHz (2.48–2.63 kHz).

Distribution and habits. *Boulenophrys lushanensis* sp. nov. is currently known only from its type locality, Mount Lushan, Jiujiang City, Jiangxi Province, China. It is distributed across elevations of 200 to 1200 m a.s.l., from lowland subtropical evergreen broadleaved forests to upland mixed coniferous–broadleaved forests, inhabiting the forest floor, leaf litter near montane streams, and roadside drainage channels.

**Figure 5:**
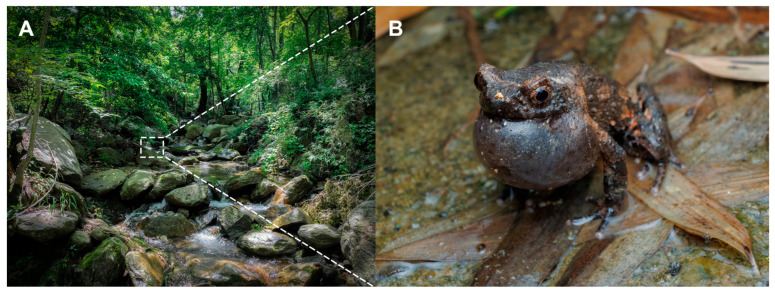
Habitat of *Boulenophrys lushanensis* sp. nov. at the type locality: (**A**) Stream habitat within low-elevation evergreen forest; (**B**) calling male paratype (JXNU 21042802) perched on a rock.

From April to July, males can be heard calling on rocks in the stream or beneath accumulated coniferous leaf litter (Figure 5). Tadpoles are present throughout the year, and tadpoles of the stone frog (*Quasipaa spinosa*) were also recorded co-occurring in the same aquatic habitats.

### 3.3. Comparisons

Key diagnostic characters distinguishing the new species from all recognized *Boulenophrys* congeners are presented in Table 2.

*Boulenophrys lushanensis* sp. nov. is phylogenetically closest to *B. jinggangensis* and forms a sister clade with it. The new species can be unambiguously distinguished from *B. jinggangensis* by the following combination of characters: (1) larger male body size, SVL 40.5–44.7 mm (vs. 35.1–36.7 mm); (2) heels not meeting when flexed hindlimbs are held at right angles to the body axis (vs. heels meeting); (3) smaller horn-like tubercle at upper eyelid (vs. large tubercle); (4) relative finger lengths: I < II < IV < III (vs. II < I < IV < III). Regarding call characteristics, the new species differs from *B. jinggangensis*, as shown in Table 3 (Audio S1): it has a slower call repetition rate, 1.27 call/s (vs. 6.07 call/s), has a longer call group durations and inter-call intervals, 267.6–326.6 ms and 644.5–969.2 ms (vs. 100.3–113.1 ms and 140.1–222.9 ms), has lower dominant frequency, 2.48–2.63 kHz (vs. 3.06–3.75 kHz), and more pulses per call group, 41–50 (vs. 7–16). The waveform and spectrogram of a 1.7-s call segment for the two species are shown in Figure 4.

Additionally, *Boulenophrys lushanensis* sp. nov. can easily be distinguished from the following congeners by its vomerine teeth present: *B. acuta*, *B. angka*, *B. anlongensis*, *B. baishanzuensis*, *B. baolongensis*, *B. binchuanensis*, *B. binlingensis*, *B. boettgeri*, *B. brachykolos*, *B. caobangensis*, *B. congjiangensis*, *B. changyangensis*, *B. cheni*, *B. chishuiensis*, *B. daoji*, *B. dupanglingensis*, *B. gaolanensis*, *B. gutu*, *B. huangniushiensis*, *B. hengshanensis*, *B. hungtai*, *B. jiangi*, *B. kuatunensis*, *B. leishanensis*, *B. lushuiensis*, *B. lini*, *B. lishuiensis*, *B. minor*, *B. mirabilis*, *B. mufumontana*, *B. dalaolingensis, B. daxuemontis*, *B. obesa*, *B. ombrophila*, *B. pepe*, *B. sanmingensis*, *B. shuichengensis*, *B. spinata*, *B. shunhuangensis*, *B. tuberogranulatus*, *B. wugongensis*, *B. wuliangshanensis*, *B. wushanensis*, *B. xiangnanensis*, *B. xianjuensis*, *B. xuefengmontis*, *B. yaoshanensis*, *B. yangmingensis*, *B. yunkaiensis* (vs. vomerine teeth absent).

*Boulenophrys lushanensis* sp. nov. can be distinguished from the following congeners by its heels not meeting when flexed hindlimbs held at right angles to body axis: *B. daiyunensis*, *B. yingdeensis* (vs. heels just meeting or overlapping), from *B. jingdongensis*, *B. jiulianensis*, *B. jinggangensis*, *B. liboensis*, *B. nanlingensis*, *B. omeimontis*, *B. qianbeiensis*, *B. palpebralespinosa*, *B. sangzhiensis*, *B. shimentaina*, *B. tongboensis*, *B. yezhongensis* (vs. heels just meeting), and from *B. caudoprocta*, *B. fanjingmontis*, *B. elongata* (vs. heels overlapping).

For the remaining, *Boulenophrys lushanensis* sp. nov. can be distinguished from *B. dongguanensis*, *B. fengshunensis*, *B. insularis*, *B. lichun*, *B. nankunensis*, *B. puningensis*, *B. hoanglienensis*, *B. fansipanensis*, *B. frigida*, *B. daweimontis* by its lateral fringes on toes narrow (vs. lateral fringes absent), and from *B. rubrimera* by its rudimentary webbing on toes (vs. lacking webbing).

## 4. Discussion

Phylogenetically, our analyses based on concatenated mitochondrial genes recover *Boulenophrys lushanensis* sp. nov. as a distinct evolutionary lineage, forming a well-supported sister clade to *B. jinggangensis*. The uncorrected COI p-distance between the new species and *B. jinggangensis* falls within the range of interspecific divergence observed in established cases of recently diverged amphibian sister taxa and is consistent with previously published interspecific distances within *Boulenophrys* (see Table S3). While COI divergence provides useful comparative information, thresholds for mitochondrial markers are taxon-dependent; conservative thresholds vary and should be treated cautiously, and single-locus distances alone are not definitive evidence of species status. We therefore interpret the molecular evidence in an integrative framework. The concordance of (1) a well-supported mitochondrial clade for the Lushan lineage; (2) a COI divergence consistent with interspecific comparisons in closely related taxa; (3) marked and consistent morphological differences (body size, eyelid tubercle, finger formula); and (4) striking, quantitative distinctions in advertisement calls together provide mutually reinforcing lines of evidence for species-level differentiation. At the same time, we explicitly acknowledge the limitations of mitochondrial-only inference: mitochondrial markers can be misleading due to incomplete lineage sorting, recent divergence, or introgression, and tree topology/distance from mtDNA may not always reflect nuclear differentiation. Accordingly, we recommend additional sampling of independent nuclear loci (or target UCE/USCO loci or SNP data) and the application of coalescent-based species delimitation methods to further test the hypothesis of species status. Until such data are available, the congruence among mitochondrial, morphological and acoustic evidence supports the recognition of *B. lushanensis* sp. nov.

*Boulenophrys lushanensis* sp. nov. described from Mount Lushan, is a relatively young fault-block massif formed after the late Himalayan orogeny about 20 million years ago. Subsequently, driven by the combined stresses of the Pacific plate and the Himalayan orogeny, Miocene and later uplifts shaped its steep ridges, cliffs and glaciated valleys [20]. Within the massif, heavy rainfall and glacial melt give rise to numerous streams, which carve waterfalls, lakes and deep forest valleys, resulting in a complex mosaic of riparian microhabitats. Moreover, surrounded by the Yangtze River, Poyang Lake water systems and broad plains, gene flow between Mount Lushan’s amphibians and outside populations is further limited [21]. Species of *Boulenophrys* are known for their limited dispersal ability and dependence on specific aquatic habitats. They typically occur in topographically and climatically diverse regions, which have not only contributed to the genus’s remarkable species diversity but also led to many species being restricted to narrow geographic ranges [22,23,24]. Consequently, under classic allopatric models [25], such geographic isolation and specialized montane stream habitats in Mount Lushan are expected to promote rapid lineage divergence within *Boulenophrys*.

The discovery of this new species highlights the previously overlooked diversity in the isolated mountain systems of northern Jiangxi Province in central China. The addition of *B. lushanensis* sp. nov. brings the total number of *Boulenophrys* species recorded in Jiangxi to 13. This finding suggests that the herpetofauna of this area is more diverse than historically appreciated and warrants further exploration. We consider that one of the most significant morphological differences between the new species and its phylogenetically closest relative, *B. jinggangensis*, is body size. Adult males of the new species (SVL 40.5–44.7 mm) are even larger than adult females of *B. jinggangensis* (SVL 38.4–41.6 mm), despite some overlap in their size ranges. This suggests that SVL should be considered an important diagnostic trait in species identification, particularly among closely related species. In addition, the advertisement calls of the two species differ markedly, making them readily distinguishable by ear. The present acoustic analyses are based on a limited sample size, and additional data from more individuals will help to further confirm this pattern. Thus, further studies combining detailed morphological comparisons, phylogenetic analyses and acoustic analyses are recommended to clarify species boundaries within the genus. Moreover, continued field surveys in northern Jiangxi are essential to fully document the true diversity and evolutionary history of *Boulenophrys* in this biogeographically significant region.

## 5. Conclusions

The integrative evidence from molecular phylogenetics, detailed morphology, and acoustic analyses supports the recognition that the Mount Lushan population, long regarded as *Boulenophrys boettgeri*, represents a distinct species: *Boulenophrys lushanensis* sp. nov. Continued surveys and taxonomic revisions in northern Jiangxi are likely to reveal further cryptic diversity and contribute to a better understanding of amphibian evolution in this biogeographically complex region.

## Figures and Tables

**Table 2:** Key diagnostic characters for all 77 *Boulenophrys* species.

Species		SVL in Males (in mm)	SVL in Females (in mm)	Horn-Like Tubercle at Upper Eyelid: Larger (T); Smaller (F)	Vomerine Teeth: Present (T); Absent (F)	Tongue: Notched(T); Not Notched (F)	Heels Over- Lapping: (T) Just Meeting (N); Not Meeting (F)	Lateral Fringes on Toes: Wide (T); Narrow (N); Absent (F)	Webs on Toes: One-Fourth (T); Rudimentary(N); Lacking (F)
1	*B. lushanensis* sp. nov.	40.5–44.7	46.0–50.2	F	T	F	F	N	N
2	*B. acuta*	27.1–33.0	28.1–33.6	T	F	F	F	N	N
3	*B. angka*	31.2–32.1	37.5–39.2	F	F	F	T/N	F	N
4	*B. anlongensis*	40.0–45.5	48.9–51.2	F	F	F	T	N	N
5	*B. baishanzuensis*	28.4–32.4	/	F	F	F	T	N	F
6	*B. baolongensis*	42.0–45.0	/	F	F	T	N	F	F
7	*B. binchuanensis*	32.0–36.0	40.2–42.5	F	F	T/F	N	T	N
8	*B. binlingensis*	45.1–51.0	/	F	F	T	T	/	N
9	*B. boettgeri*	34.5–37.8	39.7–46.8	F	F	T	N	T	N
10	*B. brachykolos*	33.7–39.3	33.9–45.9	F	F	F	F	F	N
11	*B. caobangensis*	34.9–38.9	/	F	F	F	/	F	N
12	*B. caudoprocta*	81.3	/	T	T	F	N	/	N
13	*B. congjiangensis*	28.6–33.4	38.4–40.2	F	F	F	T	N	N
14	*B. changyangensis*	39.4–43.3	/	F	F	F	T	F	F
15	*B. cheni*	26.2–29.5	31.8–34.1	F	F	T	T	T	N
16	*B. chishuiensis*	43.4–44.1	44.8–49.8	F	F	F	T	F	N
17	*B. daiyunensis*	27.6–28.7	33.7–35.6	F	T	F	T/N	N	N
18	*B. daoji*	32.6–33.6	37.5–41.4	F	F	F	F	N	N
	*B. dalaolingensis*	49.9–56.2	50.3–60.0	F	F	T	T	T	T
19	*B. fanjingmontis*	58.2–63.6	62.8–72.2	F	T	T	N	T	N
20	*B. dongguanensis*	30.2–39.3	/	F	T	F	F	F	N
21	*B. dupanglingensis*	37.8–40.2	41.8–45.9	F	F	F	T	F	N
22	*B. elongata*	28.2–28.5	35.1–37.6	F	T	F	N	N	F
23	*B. hoanglienensis*	37.4–47.6	59.6	F	T	T	/	F	N/F
	*B. huangnniushienensis*	37.9–40.7	/	F	F	F	T/N	F	F
24	*B. fansipanensis*	30.9–44.3	41.7–42.5	F	T	T/F	/	F	F
25	*B. fengshunensis*	34.3–39.4	42.5–44.9	F	T	F	F	F	N
26	*B. frigida*	30.3–31.8	/	F	T	T/F	/	F	F
27	*B. gaolanensis*	29.2–30.9	32.0–34.9	F	F	F	F	F	F
	*B. gutu*	34.4–38.0	48.4	F	F	F	F	N	N
28	*B.hengshanensis*	35.7–41.2	37.5–50.2	F	F	F	F	F	F
29	*B. jingdongensis*	53.0–56.5	63.5	F	T	T	T	T	T
30	*B. hungtai*	25.8–33.3	/	F	F	F	F	F	F
31	*B. insularis*	36.8–41.2	47.1	F	T	T	F	F	N
32	*B. jiangi*	34.4–39.2	39.5–40.4	F	F	F	T	F	N
33	*B. jiulianensis*	30.4–33.9	34.1–37.5	F	T	T	T	F	N
34	*B. jinggangensis*	35.1–36.7	38.4–41.6	T	T	F	T	N	N
35	*B. liboensis*	60.5–67.7	60.8–70.6	T	T	T	T	T	N
36	*B. kuatunensis*	26.2–31.4	26.6–37.3	F	F	T	T/F	F/N	F
37	*B. leishanensis*	30.4–38.7	42.3	F	F	F	T	F	N
38	*B. lichun*	33.5–37.0	47.1	F	T	F	F	F	F
39	*B. lushuiensis*	31.0–34.8	/	F	F	T	/	N	N
40	*B. lini*	34.1–39.7	37.0–39.9	F	F	F	T	T	N
41	*B. lishuiensis*	30.7–34.7	36.9–40.4	F	F	F	/	F	F
42	*B. minor*	34.5–41.2	/	F	F	T	T/N	F	N
43	*B. nanlingensis*	30.5–37.3	/	F	T	T	T	N	N
44	*B. mirabilis*	55.8–61.4	68.5–74.8	T	F	F	T	N	N
45	*B. mufumontana*	30.1–30.8	36.3	F	F	F	T	N	N
46	*B. nankunensis*	29.9–34.9	39.4–41.9	F	T	F	F	F	N
47	*B. omeimontis*	56.0–59.5	68.0–72.5	F	T	T	T	N	N
48	*B. daxuemontis*	41.2–46.2	51.8–58.6	F	F	F	T	N	N
49	*B. obesa*	35.6	37.5–41.2	F	F	F	F	F	N
50	*B. ombrophila*	27.4–34.5	32.8–35.0	F	F	F	F	F	F
51	*B. qianbeiensis*	49.3–58.2	/	F	T	T	T	T	T
52	*B. palpebralespinosa*	36.2–38.0	/	T	T	F	T	T	T
53	*B. pepe*	35.3–36.4	/	F	F	T	F	F	F
54	*B. puningensis*	31.7–34.6	37.8–38.3	F	T	F	F	F	N
55	*B. sangzhiensis*	54.7	/	F	T	T	T	N	N
56	*B. rubrimera*	26.6–30.8	/	F	T	T/F	/	N	F
57	*B. sanmingensis*	27.0–29.5	29.5	F	F	T	T	T	N
58	*B. shuichengensis*	102.0–118.3	99.8–115.6	T	F	T	/	T	T
59	*B. shimentaina*	28.0–30.6	/	F	T	F	T	N	N
60	*B. spinata*	47.2–54.4	54.0–55.0	F	F	T	T	T	T
61	*B. shunhuangensis*	30.3–33.7	37.6	F	F	F	T	F	F
62	*B. tongboensis*	26.5–31.5	/	F	T	T	T	F	F
63	*B. daweimontis*	34.0–37.0	40.0–46.0	F	T	/	/	F	F
64	*B. tuberogranulatus*	33.2–39.0	50.5	F	F	F	/	F	N
65	*B. wugongensis*	31.0–34.1	38.5–42.8	F	F	F	F	F	N
66	*B. wuliangshanensis*	27.3–31.6	41.3	F	F	T/F	T	F	F
67	*B. wushanensis*	30.4–35.5	38.4	F	F	F	F	F/T	T
68	*B. xiangnanensis*	38.6–42.0	44.4	F	F	F	N	T	N
69	*B. xianjuensis*	31.0–36.3	41.6	F	F	F	T	N	N
70	*B. xuefengmontis*	37.0–38.3	45.3–48.9	F	F	F	T/N	F	F
71	*B. yaoshanensis*	32.5–42.6	46.6–47.4	F	F	F	T/N	F	N
72	*B. yangmingensis*	33.2–37.1	45.2	F	F	F	T	N	N
73	*B. yezhongensis*	41.2–46.2	51.8–58.6	F	T	F	T	N	N
74	*B. yingdeensis*	33.2–35.3	36.3–45.8	F	T	F	T/N	F	N
75	*B. yunkaiensis*	35.3–40.0	45.3–46.1	F	F	F	T/N	F	N

‘/’ for missing data.

**Table 3:** Comparisons of characteristics of advertisement calls of *Boulenophrys lushanensis* sp. nov. and *B. jinggangensis*.

Call Character	*Boulenophrys lushanensis* sp. nov.			*Boulenophrys jinggangensis*
	Unvouchered	JXNU21042803	JXNU21042802	Unvouchered
Number of call groups measured	9	6	7	9
Call duration (ms)	290.6 ± 15.6 (271.3–322.0)	301.4 ± 12.4 (286.9–322.3)	298.2 ± 18.1 (267.6–326.6)	107.0 ± 4.6 (100.3–113.1)
Intercall interval (ms)	737.7 ± 112.6 (623.4–960.3)	834.4 ± 114.5 (634.3–960.5)	790.0 ± 124.3 (644.5–969.2)	165.1 ± 27.1 (140.1–222.9)
Call repetition rate (calls/s)	1.36	1.2	1.27	6.07
Pulses/call	33–53	51–59	41–50	7–16
Dominant frequency (kHz)	2.54 ± 0.03 (2.50–2.58)	2.64 ± 0.04 (2.56–2.68)	2.56 ± 0.06 (2.48–2.63)	3.28 ± 0.30 (3.06–3.75)

## Data Availability

The original contributions presented in the study are included in the article/Supplementary Materials, further inquiries can be directed to the corresponding author.

## Supplementary Materials

The following supporting information can be downloaded at: https://www.mdpi.com/article/10.3390/ani15213197/s1, Table S1: Literature for morphological characters of 77 recognized species of *Boulenophrys* [26,27,28,29,30,31,32,33,34,35,36,37,38,39,40,41,42,43,44,45,46,47,48,49,50,51,52,53,54,55,56,57,58,59,60,61,62,63,64,65]; Table S2: Sample list of genus *Boulenophrys* and outgroups for phylogenetic analysis in this study; Table S3: Uncorrected p-distance (%) among *Boulenophrys* species from the COI; Audio S1: Advertisement call recordings of *Boulenophrys lushanensis* sp. nov. and *Boulenophrys jinggangensis*.
